# Transcatheter tricuspid valve repair: early experience in the Netherlands

**DOI:** 10.1007/s12471-021-01613-3

**Published:** 2021-08-20

**Authors:** F. Meijerink, K. T. Koch, R. J. de Winter, M. Holierook, B. J. W. M. Rensing, L. Timmers, F. D. Eefting, M. J. Swaans, B. J. Bouma, J. Baan

**Affiliations:** 1 Department of Cardiology, Amsterdam UMC, location AMC, Amsterdam, The Netherlands; 2 Department of Cardiology, St Antonius Hospital, Nieuwegein, The Netherlands

**Keywords:** Tricuspid regurgitation, Transcatheter treatment, Valvular heart disease, Echocardiography

## Abstract

**Background:**

Symptomatic tricuspid regurgitation (TR) is increasingly prevalent and impairs quality of life and survival, despite medical treatment. Transcatheter tricuspid valve repair (TTVR) has recently become available as a treatment option for patients not eligible for tricuspid valve surgery. In this study we describe the early experience with TTVR in the Netherlands.

**Methods:**

All consecutive patients scheduled for TTVR in two tertiary hospitals were included in the current study. Patients were symptomatic and had severe functional TR. TTVR was performed either with the MitraClip (off-label use) or dedicated TriClip delivery system and device. Procedural success was defined as achievement of clip implantation, TR reduction ≥ 1 grade and no need for re-do surgical or transcatheter intervention. Clinical improvement was evaluated after 4 weeks.

**Results:**

Twenty-one patients (median age 78 years, 33% male, 95% New York Heart Association class ≥ 3, 100% history of atrial fibrillation) underwent TTVR. Procedural success was achieved in 16 patients, of whom 15 reported symptomatic improvement (New York Heart Association class 1 or 2). There was no in-hospital mortality and no major complications occurred. Baseline glomerular filtration rate and TR coaptation gap size were associated with procedural success.

**Conclusion:**

The current study showed that TTVR seems a promising treatment option for patients with severe functional TR deemed high risk for surgery. Successful TR reduction is most likely in patients with limited coaptation gap size and strongly determines clinical benefit. Adequate patient selection and timing of treatment seem essential for an optimal patient outcome.

**Supplementary Information:**

The online version of this article (10.1007/s12471-021-01613-3) contains supplementary material, which is available to authorized users.

## What’s new?

- Transcatheter tricuspid valve repair (TTVR) is now available for patients with symptomatic tricuspid regurgitation (TR) not eligible for valve surgery.
- When TR reduction ≥ 1 grade can be established, clinical improvement is likely.
- Coaptation gap size is the most important determinant of a successful procedure.
- Patients with no significant left-sided heart valve disease, preserved right ventricular function, no pulmonary hypertension and a coaptation gap size < 10 mm should be considered for TTVR.

## Introduction

Tricuspid regurgitation (TR) is as common as mitral regurgitation (MR) in the general population and its prevalence increases with age. Functional TR accounts for up to 90% of patients. Annular dilatation and increased tricuspid leaflet tethering in relation to right ventricular (RV) pressure and/or volume overload cause functional TR. Left-sided heart disease, atrial fibrillation (AF) or pulmonary hypertension are frequently involved in the pathogenesis of TR [1, 2]. Significant TR often leads to right-sided heart failure symptoms and is associated with increased morbidity and mortality [3]. Once TR is present, a vicious circle arises where TR begets more RV and right atrial (RA) dilatation, leading to more TR. Whilst tricuspid valve (TV) intervention is indicated when symptomatic TR is present, the risk of surgery is deemed to be too high in most patients with isolated TR due to the presence of RV dysfunction, advanced age or other (cardiac) co-morbidities [4]. Until recently, these patients could only be treated with medical therapy, but transcatheter tricuspid valve repair (TTVR) is now available. The aim of this study was to evaluate the early experience with TTVR in the Netherlands with a focus on patient selection, safety and effectiveness of the procedure as well as determinants of procedural success.

## Methods

### Study population

All consecutive patients who underwent TTVR at Amsterdam University Medical Centre, location AMC, Amsterdam and St Antonius Hospital, Nieuwegein between October 2019 and February 2021 were included. Patients were admitted via the heart team and screened by a dedicated transcatheter valve intervention team. In all patients, the risk of TV surgery was deemed to be too high. All patients consented to participation and the study was conducted in accordance with the Declaration of Helsinki.

### Echocardiography

TR severity was determined by transthoracic echocardiography (TTE) and graded according to the latest classification, including massive and torrential TR [5]. These additional grades are highly relevant, because patients often present with TR largely exceeding the guideline cutoff criteria for severe TR. Reduction to less than severe TR is not always achieved, but has appeared to be associated with improved outcome. Standard parameters were included according to the guidelines of the American Society for Echocardiography (ASE) and European Association of Cardiovascular Imaging (EACVI) [6, 7].

### Procedure

**Fig. 1:**
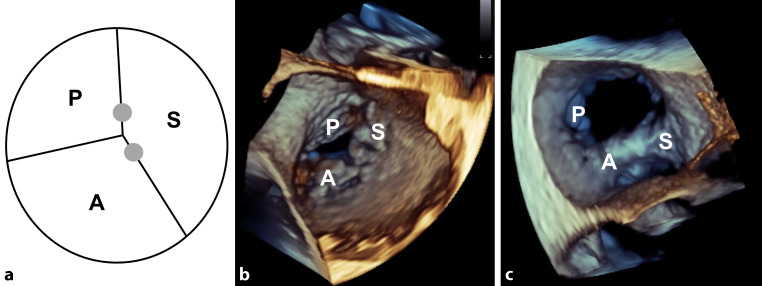
**a** Tricuspid valve (TV) anatomy and most common clip locations (*grey dots*). Three-dimensional echocardiographic view of the TV **b** before and **c** after implantation of two clips on the anterior and septal leaflets. *A* anterior leaflet, *P* posterior leaflet, *S* septal leaflet

TTVR was performed using the clip-based edge-to-edge technique with the MitraClip device (Abbott, Santa Clara, CA, USA) as off-label use in 13 patients. The dedicated TriClip device (Abbott) has been available for clinical use in the Netherlands since September 2020 and was used in 8 patients [8]. Major improvements offered by the TriClip device include (1) extended flexion of the guiding catheter, which provides improved height adjustment above the valve and (2) improved motion in septal and lateral directions, which increases the reach when placing clips in the antero-septal (AS) and postero-septal (PS) commissures, allowing more complex jets to be treated. Figure S1 (see Electronic Supplementary Material) shows both systems and the specific differences. After obtaining access to the femoral vein, the guiding catheter was introduced. The clip delivery system was subsequently inserted into the guiding catheter and positioned above the TV. The principle of the edge-to-edge technique is to grasp two leaflets with the clip, closing and deploying the device and thereby creating two or more orifices [9]. The grasp was targeted at either the AS, PS or antero-posterior leaflets (Fig. 1). If TR reduction was not sufficient after implantation of one clip, additional clips were implanted, depending on the TV gradient. The procedures were done with the patient under general anaesthesia, guided by three-dimensional transoesophageal echocardiography (TEE) and fluoroscopy. The treatment team consisted of one imaging cardiologist and two interventional cardiologists, who together determined the clip strategy.

### Data and outcome

Baseline clinical and echocardiographic data were collected. Clinical follow-up was conducted after 4 weeks. Technical success was achieved when the clip could be implanted and procedural success was defined as a successful clip implantation, TR reduction ≥ 1 grade and no need for additional surgical or transcatheter TV intervention at follow-up. Procedural and in-hospital complications were recorded. Comparisons were made between patients that underwent successful and non-successful procedures.

Retrospective stratification of eligibility for tricuspid edge-to-edge repair was performed for each patient according to anatomical and functional criteria (Tab. 1; [10]).

**Table 1:** Anatomical and functional criteria for transcatheter tricuspid edge-to-edge repair (adapted from Hausleiter et al. [10], including number of patients)

	Most appropriate	*n*	Possibly appropriate	*n*	Least appropriate	*n*
Leaflet appearance	Normal	20	Primary TR with prolapse (width < 12 mm)	0	Thickening, shortening, destruction or large prolapse (> 12 mm)	1
Coaptation gap	< 4 mm	6	4–7 mm	3	> 7 mm	12
Jet location	Central AS commissure	20	Central PS commissure	0	Non-central AP commissure	1
Leaflet visualisation (echo)	Good	17	Sufficient	4	Insufficient	0
PM/ICD lead	No	21	Present No interaction	0	Lead-induced TR	0
RV function	Normal	10	Moderately reduced	10	Severely reduced	1
Systolic PAP	Normal	16	40–60 mm Hg	5	> 60 mm Hg	0

*AP* antero-posterior, *AS* antero-septal, *ICD* implantable cardioverter defibrillator, *PAP* pulmonary artery pressure, *PM* pacemaker, *PS* postero-septal, *RV* right ventricle, *TR* tricuspid regurgitation

### Statistical analysis

Categorical variables are expressed as *n* (%) and were compared using the chi-squared or Fisher’s exact test. Continuous variables are expressed as median and range (minimum to maximum) and were compared using the Mann-Whitney U test. Statistical significance was established at *p* < 0.05. All analyses were done using SPSS version 26 (IBM, Armonk, NY, USA).

## Results

### Study population

Twenty-one patients underwent TTVR. Clinical data of all patients are presented in Tab. 2. Twenty patients (95%) were in New York Heart Association (NYHA) class 3 or 4. Most common symptoms were dyspnoea (95%), peripheral oedema (62%) and ascites (19%); 13 patients (62%) had recently been hospitalised for heart failure. All patients were on optimal doses of medical therapy for heart failure: 91% loop diuretic, 76% beta-blocker, 57% aldosterone antagonist, 38% angiotensin converting enzyme inhibitor/angiotensin II antagonist. All patients had functional TR (38% severe, 43% massive, 19% torrential).

**Table 2:** Baseline characteristics

Variable	All patients (*n* = 21)	Procedural success (*n* = 16)	No procedural success (*n* = 5)	*p*-value
*Clinical*				
Age (years)	78 (60–87)	79 (60–87)	76 (60–84)	0.36
Male (%)	7 (33)	5 (31)	2 (40)	1.00
BMI (kg/m^2^)	24.9 (18.4–36.1)	24.5 (20.2–36.1)	25.1 (18.4–27.1)	0.80
COPD (%)	1 (5)	0 (0)	1 (20)	0.24
Peripheral arterial disease (%)	0 (0)	0 (0)	0 (0)	–
Stroke	4 (19)	4 (25)	0 (0)	0.53
Diabetes mellitus	4 (19)	2 (13)	2 (40)	0.23
Atrial fibrillation	21 (100)	16 (100)	5 (100)	–
Myocardial infarction	1 (5)	0 (0)	1 (20)	0.24
Pacemaker/ICD	0 (0)	0 (0)	0 (0)	–
*Previous cardiac surgery*				0.74
CABG	1 (5)	1 (6)	0 (0)	
AVR	2 (10)	2 (13)	0 (0)	
MVR	2 (10)	1 (6)	1 (20)	
Pericardectomy	2 (10)	1 (6)	1 (20)	
ASD closure	1 (5)	1 (6)	0 (0)	
GFR (ml/min per 1.73 m^2^)	58 (13–83)	51 (13–80)	77 (53–83)	0.04
*NYHA class*				0.85
2	1 (5)	1 (6)	0 (0)	
3	16 (76)	12 (75)	4 (80)	
4	4 (19)	3 (19)	1 (20)	
*Echo*				
LVEF (%)	50 (32–71)	50 (40–71)	50 (32–54)	0.32
LVEDV (ml)	72 (44–180)	68 (44–180)	78 (52–90)	0.36
LVESV (ml)	35 (20–95)	33 (20–95)	44 (25–60)	0.25
LA volume (ml)	81 (37–271)	80 (37–271)	100 (72–195)	0.40
RA volume (ml)	134 (70–678)	123 (70–273)	187 (70–678)	0.22
TAPSE (mm)	13 (8–26)	13 (8–26)	16 (11–22)	0.46
RV S’ (cm/s)	8 (5–15)	8 (5–15)	9 (7–10)	0.78
Systolic PAP (mm Hg)	35 (20–51)	31 (20–42)	44 (35–51)	0.06
MR ≥ moderate (%)	6 (29)	5 (31)	1 (20)	1.00
AS (%)	1 (5)	0 (0)	1 (20)	0.24
*TR grade*				0.43
3 (severe)	8 (38)	6 (38)	2 (40)	
4 (massive)	9 (43)	6 (38)	3 (60)	
5 (torrential)	4 (19)	4 (25)	0 (0)	
TR vena contracta (mm)	14 (2–25)	14 (2–25)	13 (8–19)	0.90
TR coaptation gap (mm)	8 (1–20)	7 (1–13)	14 (7–20)	0.01
Functional TR	21 (100)	16 (100)	5 (100)	–
Annulus diameter (mm)	48 (35–68)	47 (35–56)	50 (40–68)	0.56

Continuous variables are expressed as median (range). For categorical variables number (%) are shown

*AS* aortic valve stenosis, *ASD* atrial septal defect, *AVR* aortic valve replacement, *BMI* body mass index, *CABG* coronary artery bypass grafting, *COPD* chronic obstructive pulmonary disease, *GFR* glomerular filtration rate, *ICD* implantable cardioverter defibrillator, *LA* left atrium, *LVEDV* left ventricular end-diastolic volume, *LVESV* left ventricular end-systolic volume, *LVEF* left ventricular ejection fraction, *MR* mitral regurgitation, *MVR* mitral valve repair/replacement, *NYHA* New York Heart Association, *PAP* pulmonary artery pressure, *RA* right atrium, *RV S’* right ventricular systolic myocardial velocity, *TAPSE* tricuspid annular plane systolic excursion, *TR* tricuspid regurgitation

### Procedural characteristics, complications and outcome

In Tab. 3 procedural and outcome data are shown. Technical success was achieved in 18 patients (86%) and TR could be reduced (≥ 1 grade) in 17 patients (81%). No in-hospital mortality occurred. Five patients experienced a femoral access-site bleeding, which could be conservatively managed with a pressure bandage in 4 patients. In 1 patient the femoral vein was inadvertently punctured, resulting in a lesion of the femoral vein after introduction of the guiding catheter. Surgical exploration including femoral vein repair was needed after the procedure.

**Table 3:** Procedural characteristics and outcome in hospital and at follow-up

Variable	All patients (*n* = 21)	Procedural success (*n* = 16)	No procedural success (*n* = 5)	*p*-value
*Procedural*				
Delivery system				1.00
MitraClip	13 (62)	10 (63)	3 (60)	
TriClip	8 (38)	6 (37)	2 (40)	
Technical success	18 (86)	16 (100)	2 (40)	0.008
TR reduction ≥ 1 grade	17 (81)	16 (100)	1 (20)	0.001
TV mean gradient (mm Hg)	2 (1–5)	2 (1–5)	3 (3–3)	0.13
Number of clips	2 (0–4)	2 (1–4)	0 (0–3)	0.17
Clip location (leaflets)				0.32
Antero-septal	9 (43)	9 (56)	2 (40)	
Antero-septal/Postero-septal	7 (33)	5 (31)	0 (0)	
Antero-septal/Antero-posterior	1 (5)	1 (6)	0 (0)	
Postero-septal	1 (5)	1 (6)	0 (0)	
*Safety*				
Death	2 (10)	1 (6)	1 (20)	0.43
Femoral access-site bleeding	5 (24)	4 (25)	1 (20)	1.00
Single leaflet detachment	2 (10)	0 (0)	2 (40)	0.05
*Outcome*				
TR reduction				< 0.001
≥ 2 grades	10 (48)	10 (63)	0 (0)	
1 grade	7 (33)	6 (38)	1 (20)	
No reduction	4 (19)	0 (0)	4 (80)	
TR grade ≤ moderate at discharge	12 (57)	12 (75)	0 (0)	0.006
NYHA class 1 or 2 at follow-up	15 (71)	15 (94)	0 (0)	0.001
Surgical or percutaneous re-do procedure	3 (14)	0 (0)	3 (60)	0.008

Continuous variables are expressed as median (range). For categorical variables number (%) are shown

*NYHA* New York Heart Association, *TR* tricuspid regurgitation, *TV* tricuspid valve

None of the patients experienced myocardial infarction, renal failure, pulmonary embolism, device thrombosis, new liver failure, tricuspid stenosis or clip embolisation. Single leaflet clip detachment occurred in 2 patients and resulted in persistent severe TR. Both needed additional intervention (1 surgical TV replacement and 1 re-do TTVR). Procedural success was achieved in 16 patients (76%), of whom 15 reported improvement of symptoms (NYHA 1 or 2). In those who did not achieve procedural success (*n* = 5), re-do TTVR was performed in 1, TV surgery in 2 and no additional treatment was performed in 2 patients, both of who had an absolute contraindication for surgery. Two patients died (6 and 8 weeks after the procedure, respectively) due to pre-existent and irreversible RV failure. In 1 of these patients a clip could not be implanted during the procedure.

In patients that underwent a successful procedure, median glomerular filtration rate (ml/min per 1.73 m^2^) was lower (77 vs 51, *p* = 0.04) and TR coaptation gap size (mm) was smaller (14 vs. 7, *p* = 0.01). Median systolic pulmonary artery pressure (PAP) (mm Hg) was lower in patients with procedural success (44 vs. 31, *p* = 0.06), although this difference was not significant. Coaptation gap size was < 10 mm in 14 patients (93% procedural success) and > 10 mm in 7 patients (43% procedural success). The largest coaptation gap in patients that achieved procedural success was 13 mm.

### Stratification of eligibility

Tab. 1 shows seven parameters and the number of patients in each category of eligibility for TTVR. One patient with septal leaflet destruction and a large prolapse gap was classified as least eligible. Initially the patient had a massive functional TR with possible interference of a pacemaker lead. To qualify for TTVR, the lead was extracted and replaced with a leadless pacemaker. During the procedure a large prolapse segment and a short septal leaflet were seen, probably due to leaflet destruction caused by lead extraction. Twelve patients with a coaptation gap > 7 mm qualified as least eligible, 8 of whom still achieved procedural success. RV function was moderately reduced in 10 patients (qualifying as possibly eligible) and severely reduced in 1 (qualifying as least eligible). Sixteen patients had normal systolic PAP (qualifying as most eligible).

## Discussion

In this study the early experience and results of TTVR in the Netherlands are reported. Our findings demonstrate that TTVR is safe and that TR can be reduced in most patients. A successful procedure led to improvement of symptoms in > 90% of patients.

Procedural success (76% in the current study) has shown to be the main determinant of survival and clinical benefit after TTVR [11, 12]. It is important to note that moderate or severe TR often persists after a successful procedure. However the current and earlier studies confirmed that if TR could be reduced by 1 grade, clinical benefit was likely [9]. This suggests that severe, massive and torrential TR have a significant impact on clinical status, which might strongly improve after TTVR, even when only a mild reduction can be achieved. Whether this will translate to reduction of heart failure hospitalisations and better survival has yet to be confirmed. Coaptation gap size was the most relevant determinant of successful reduction in the current study. Earlier studies also mentioned leaflet tenting area (> 3.15 cm^2^), coaptation depth (> 9.75 mm) and effective regurgitant orifice area (> 69.5 mm^2^) as determinants [13]. These echocardiographic parameters should play an important role in patient selection.

Currently, no established guideline exists for TTVR and so indication for treatment is made at the patient level by a dedicated transcatheter valve intervention team. Recommendations for TTVR appropriateness, according to clinical and anatomical factors, have been made previously (Tab. 1; [10]). For successful clip implantation it is essential that adequate leaflet grasping can be performed, where the tips of two leaflets can be inserted completely into the clip device. Improved coaptation (reducing TR) and successful clip implantation (preventing single leaflet detachment) are hereby achieved. Structurally non-normal leaflets or inadequate visualisation might prevent successful leaflet grasping. Adequate TEE imaging windows of the TV are therefore highly relevant, though sometimes difficult to obtain. If a PM lead is not interfering with the leaflets, it is not a contra-indication. Recurrent TR after repair is likely in patients with severely reduced RV function and pulmonary hypertension (systolic PAP > 60 mm Hg). TTVR is therefore not recommended in such patients [10].

**Fig. 2:**
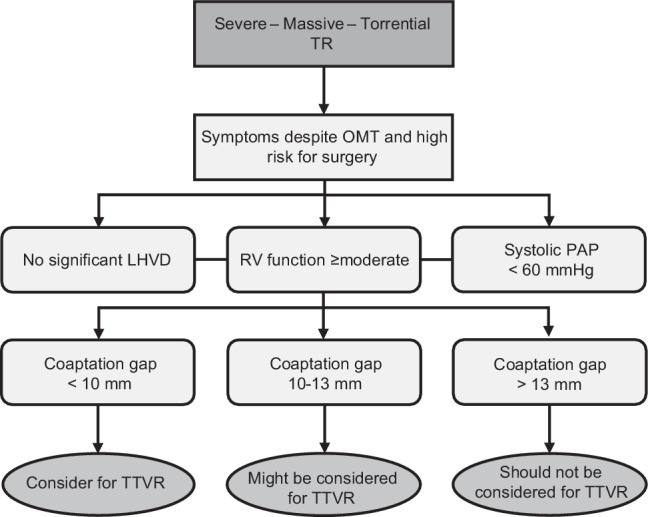
Recommendation of patients that should be referred and considered for transcatheter tricuspid valve repair (*TTVR*). *LHVD* left-sided heart valve disease, *OMT* optimal medical therapy, *PAP* pulmonary artery pressure, *RV* right ventricle, *TR* tricuspid regurgitation

After stratifying each patient for all factors, we observed that leaflet appearance and jet location were highly appropriate for TTVR in most patients (≥ 95%) and that none had a pacemaker or implantable cardioverter defibrillator lead during the procedure. The coaptation gap was > 7 mm in 12 patients, which would classify them as ‘least eligible’ according to the recommendation. However, we observed that procedural success and clinical improvement were still achieved in 8 patients with a gap size > 7 mm (largest 13 mm). It should be noted that only large clip devices (XTR, arm length 12 mm) were used, which likely explains the improved results in patients with large coaptation gaps. The recommendations by Hausleiter et al. are based on earlier experience when only small clips (NTR, arm length 9 mm) were used [14]. A recent study confirmed that patients with large coaptation gaps could be effectively treated with the XTR clip [15]. Thus, whilst TTVR is more likely to succeed when the coaptation gap is smaller, in our experience treatment was feasible with a defect of up to 10 mm, with a success rate of 93%. Three patients with a defect between 10 and 13 mm could still be effectively treated, although earlier studies report decreased success in this range [16]. RV function was classified as ‘possibly’ or ‘least’ appropriate’ in 9 patients. This did not affect procedural success, though it might affect the clinical benefit of the procedure. A recommendation for patients that could be considered for TTVR is presented in Fig. 2.

Timing of treatment for TR can be challenging. TV repair is recommended when severe TR is symptomatic or when progressive RV dysfunction is present [4]. However, once patients become symptomatic, there are often irreversible signs of right-sided heart failure and TR severity is beyond the guideline cutoff (massive or torrential). Advanced RV dilatation further distorts the TV anatomy (causing excessive leaflet tenting and a large coaptation gap) and impairs the clinical benefit after TTVR. Ideally, TTVR should be performed when the valve anatomy is not yet distorted by the disease itself and RV function is preserved. Careful diagnosis and patient evaluation, by using multi-parametric and quantitative approaches to assess TR and RV function, are therefore essential. Also, these should not be evaluated in one snapshot because varying volume status, blood pressure and heart rhythm have a great impact on assessment of the severity of TR and RV function.

As with transcatheter mitral valve repair (TMVR), complications after TTVR are rare. Single leaflet clip detachment occurred in 2 patients and prevented adequate TR reduction. It is therefore an important determinant of procedural success [9]. We did not observe TV stenosis after TTVR. Currently, a mean gradient ≤ 3 mm Hg is considered clinically acceptable [9]. A clip strategy targeted at the AS and PS leaflets warrants sufficient valve orifice area and was performed in most patients. Since tricuspid annulus dilatation is most pronounced in the anterior and posterior region, the anterior and posterior leaflets tend to become separated from the septal leaflet. Clips on both AS and PS leaflets improve coaptation and might prevent further leaflet separation [10]. Femoral access-site bleeding was seen in 5 patients and could be conservatively managed in 4. Minor access-site bleeding occurs in 5–10% of patients undergoing TMVR [17]. Haemostasis is usually achieved after removal of the guiding catheter and administration of protamine (to reverse the effect of heparin), skin closure with a figure-of-eight suture and subsequent application of a pneumatic compression device for 6 h. Oral anticoagulation should be started ≥ 24 h after the procedure. Access-site closure with ProGlide (Abbott) devices is an effective method as well [18].

### Future perspectives

TR reduction and procedural success might be further optimised by an improved (wider) clip device and delivery system allowing for independent leaflet grasping, which is expected shortly. Alternative devices might be useful for patients with large coaptation defects and extensive RV or tricuspid annulus dilatation. The positive results of TTVR pose the question as to whether a combined transcatheter MV and TV procedure could be beneficial for patients with MR and concomitant TR. Early studies showed that combined TMVR and TTVR were associated with improved clinical outcome and survival compared to TMVR alone in these patients [19, 20]. However, one could argue that the effect of TMVR on clinical status and TR should be awaited before considering TTVR.

The findings of the current study warrant further randomised studies and prospective registries to confirm the safety and effectiveness of TTVR. Ideally, such studies should include echocardiography core laboratory analysis and adequate functional assessment (exercise testing, cardiac magnetic resonance imaging) of TR and RV function before and after TTVR.

### Clinical implications

TR is a complex condition and TTVR could be an effective treatment for selected patients. The current study showed encouraging results that will hopefully translate into long-term benefit as well. One-year follow-up of the TriValve registry showed a significant decrease in hospitalisations for heart failure and improvement of symptoms, exercise capacity and quality of life. Interestingly, RV reverse remodelling was also observed [8, 21].

Procedural success has shown to be the major determinant of clinical outcome, which indicates that adequate patient selection focusing on clinical and anatomical aspects is crucial. Patients at an advanced stage of TR and RV disease might benefit less from TTVR and have a higher risk of procedural failure when a large coaptation gap is present. Timely diagnosis and treatment could therefore strongly improve outcome.

### Limitations

No independent echocardiographic data analysis was done in the current study. All echocardiographic measurements were performed by an experienced investigator (FM) and reviewed by an imaging cardiologist (BB) in accordance with the ASE and EACVI guidelines [6, 7].

## Conclusion

TTVR seems to be a promising treatment option for patients with severe functional TR deemed high risk for surgery. Successful TR reduction is most likely in patients with limited coaptation gap size and strongly determines clinical benefit. Adequate patient selection and timing of treatment seem essential for an optimal patient outcome.

## Supplementary Information

Fig. S1 MitraClip (a) and TriClip (b) device (c) as used for transcatheter tricuspid valve repair. MitraClip guiding catheter (d) has limited flexion and septo-lateral direction movements compared to the TriClip guiding catheter (e), allowing improved handling and steering mechanism and implantation of the clips (f)
