# Supplementary figures and images for: Transcatheter tricuspid valve repair: early experience in the Netherlands
Source: Neth Heart J. 2021 Aug 20;29(11):595–603. doi: 10.1007/s12471-021-01613-3 (PMC8556435; doi:10.1007/s12471-021-01613-3)

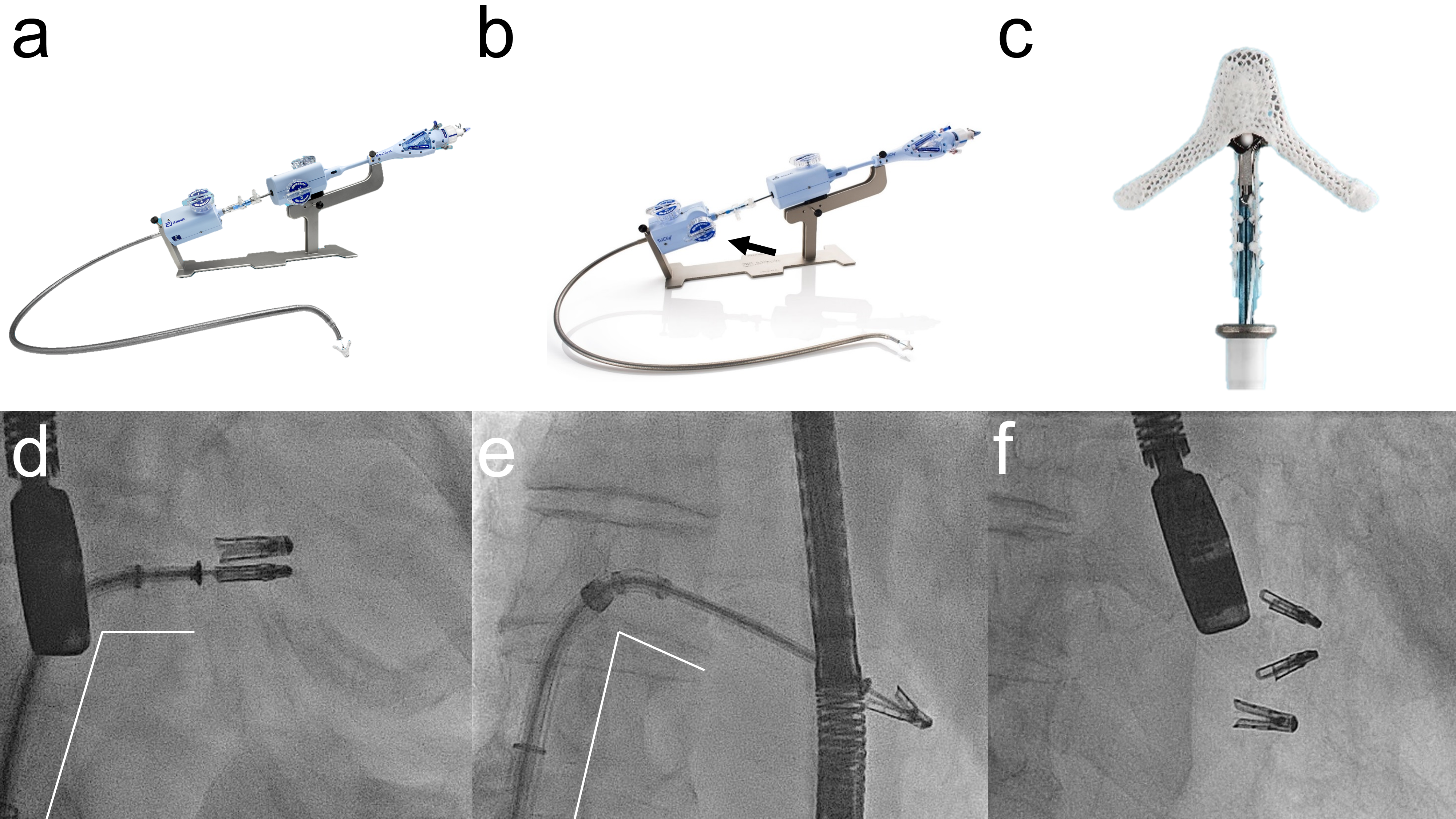

Supplement: Supplementary file 1 — Fig. S1 MitraClip (a) and TriClip (b) device (c) as used for transcatheter tricuspid valve repair. MitraClip guiding catheter (d) has limited flexion and septo-lateral direction movements compared to the TriClip guiding catheter (e), allowing improved handling and steering mechanism and implantation of the clips (f) [file 12471_2021_1613_MOESM1_ESM.tif]
